# Spinal Meningioma Surgery through the Ages—Single-Center Experience over Three Decades

**DOI:** 10.3390/medicina58111549

**Published:** 2022-10-28

**Authors:** Hanah Hadice Gull, Mehdi Chihi, Oliver Gembruch, Tobias Schoemberg, Thiemo Florin Dinger, Klaus Peter Stein, Yahya Ahmadipour, I. Erol Sandalcioglu, Ulrich Sure, Neriman Özkan

**Affiliations:** 1 Department of Neurosurgery and Spine Surgery, University Hospital Essen, University of Duisburg-Essen, 45147 Essen, Germany; 2 Center for Translational Neuro- & Behavioral Sciences (C-TNBS), University of Duisburg-Essen, 45147 Essen, Germany; 3 Department of Neurosurgery, University Hospital Magdeburg, Otto von Guericke University Magdeburg, 39120 Magdeburg, Germany

**Keywords:** spinal meningioma, meningioma, spine, surgery

## Abstract

*Background and Objectives:* Spinal meningiomas, which are well characterized and are most frequently intradural extramedullary tumors, represent 25% of all intradural spinal tumors. The goal of this study was to compare the outcomes of surgically treated patients with spinal meningiomas in two time intervals with special emphasis on postoperative functional outcomes. *Methods*: Patients with spinal meningiomas admitted to our department between 1990 and 2020 were enrolled and divided into a historic cohort (HC; treated 1990–2007) and a current cohort (CC; treated 2008–2020). Patients’ clinical data and surgical and radiological reports were retrospectively analyzed up to 5 years. Preoperative and postoperative neurological function were assessed using the modified McCormick Scale (mMCS). The Charlson Comorbidity Index (CCI) was used to evaluate the effect of comorbidities on the preoperative status and postoperative outcome. *Results:* We included 300 patients. Participants in the CC (n = 144) were significantly younger compared to those in the HC (n = 156), with twice as many patients <50 years of age (*p* < 0.001). The most common tumor location was the thoracic spine (n = 204). The median follow-up was 38.1 months (±30.3 standard deviation). A symptom duration until surgery <12 months was significantly associated with an earlier improvement in the mMCS (*p* = 0.045). In the CC, this duration was shorter and patients’ neurological function at the first and last follow-ups was significantly better than for those in the HC (*p* < 0.001 for both). *Conclusions*: Our study results suggested that the impact of surgical management and postoperative rehabilitation on spinal meningioma patients’ long-term neurological outcome has reached important milestones over the last decades. An earlier diagnosis led to earlier surgical treatment and improved patients’ postoperative neurological recovery. Our results exposed that surgical therapy for spinal meningioma should be performed within 12 months after appearance of symptoms to achieve a better recovery.

## 1. Introduction

Spinal meningiomas are rare tumors (incidence: 2–3/100,000 persons per year) that derive from the meninges of the spinal cord and represent 25–30% of all intradural extramedullary spinal tumors [1,2]. Whilst they can grow anywhere along the spine, the most frequent site is the thoracic region [3]. The peak incidence is in the sixth through eighth decade of life [4]. In contrast to cranial meningiomas, which have a male-to-female ratio of 1:2, spinal meningiomas have a ratio of 1:4, with females accounting for 75–90% of all cases [1]. Meningiomas usually are benign, slow-growing tumors [5]. The clinical symptoms are not specific and comprise pain, sensory deficits, motor weakness, and vegetative dysfunction depending on the spinal level of the tumor [6]. Surgical therapy, especially for ventrally located spinal meningiomas, may be challenging due to the location of the spinal cord [7,8,9]. Microsurgical treatment of symptomatic tumors remains the gold standard [10,11]. The postoperative outcome of spinal meningiomas is good in general, and even patients with poor preoperative neurological status can recover completely [11,12]. It remains unknown whether the improvements in postoperative outcome may be attributed to the recent technical advances in high-resolution magnetic resonance imaging (MRI), intraoperative ultrasound devices, microscopes, ultrasonic aspirators, surgical techniques, and time of surgery [13]. Therefore, we retrospectively analyzed surgically treated patients suffering from spinal meningiomas to search for possible conditions and factors that influenced the outcomes over a period of 31 years.

## 2. Materials and Methods

### 2.1. Data Collection and Analysis

This study retrospectively analyzed patients with spinal meningioma that were admitted and surgically treated between 1990 and 2020 in our neurosurgical department. Clinical data and radiological and operative reports were analyzed. The main diagnostic tool was MRI. In few cases (4.3%, n = 13) for which MRI was contraindicated, computed tomography with a contrast agent was applied.

Demographic data (age and sex), clinical characteristics (revealing symptoms, neurological status at admission, and duration of symptoms until surgery), tumor characteristics (location, histology, and recurrence), surgical therapy (approach and surgical extent of resection based on Simpson’s classification [14]), and postoperative complication were evaluated. Follow-up was performed up to 5 years and consisted of a complete neurological examination and postoperative contrast-enhanced MRI of the spine.

The modified McCormick Scale (mMCS) [15] was used to evaluate neurological function. The MCS was modified according to the current literature [16,17] to allow for a more valuable discrimination in postoperative neurological outcome. A cut-off value of mMCS > 3 was chosen because patients with a score higher than 3 suffer from severe neurological deficits with limitations in function and loss of independence; those with an mMCS ≤ 3 do not show severe neurological deficits and do not need external help. The Charlson Comorbidity Index (CCI) was calculated to assess the effect of comorbidities on the preoperative status and postoperative outcome [18]. The entire cohort was divided into two groups with approximately equal cohort sizes. A change in chairmanship in 2008 was identified as the only turning point in our department. Therefore, we compared those two different time cohorts: the historic cohort (HC) included patients treated between 1 January 1990–31 December 2007, while patients treated between 1 January 2008–31 December 2020 were allocated to the current cohort (CC) after the change in the chairmanship.

### 2.2. Ethics

This study was conducted in accordance with the Strengthening the Reporting of Observational Studies in Epidemiology (STROBE) guidelines after approval by the Institutional Review Board (Medical Faculty, University of Duisburg-Essen, Registration Number: 21-10397-BO) and followed The Code of Ethics of the World Medical Association (Declaration of Helsinki).

### 2.3. Surgical Strategy and Outcome

**Figure 1:**
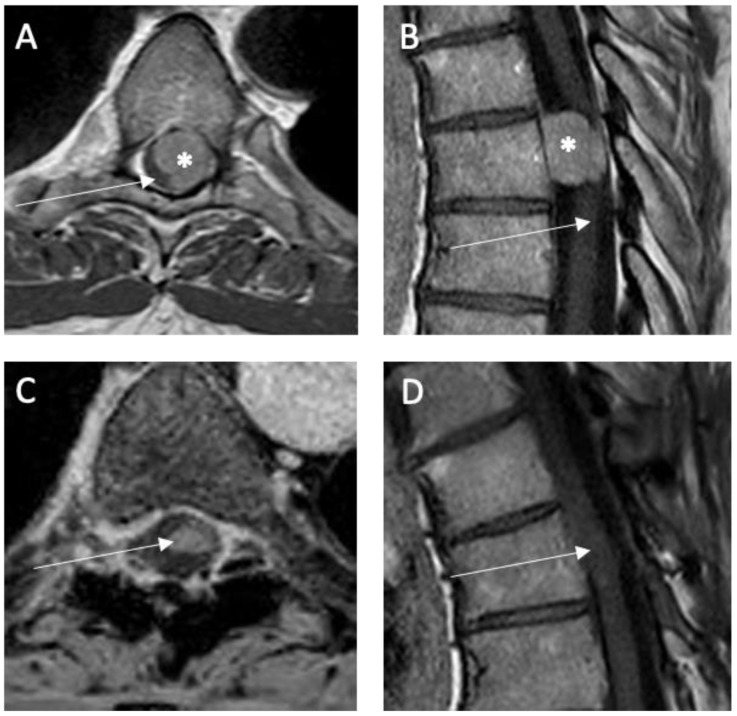
Illustrative case of a spinal meningioma (*) in thoracic level (Th 6) located ventrolaterally and left-sided relative to the spinal cord (→): preoperative T1-weighted MRI with contrast enhancement (**A**) axial and (**B**) sagittal. Postoperative T1-weighted MRI with contrast enhancement (**C**) axial and (**D**) sagittal 6 months after surgery showing no tumor rest or recurrence.

**Figure 2:**
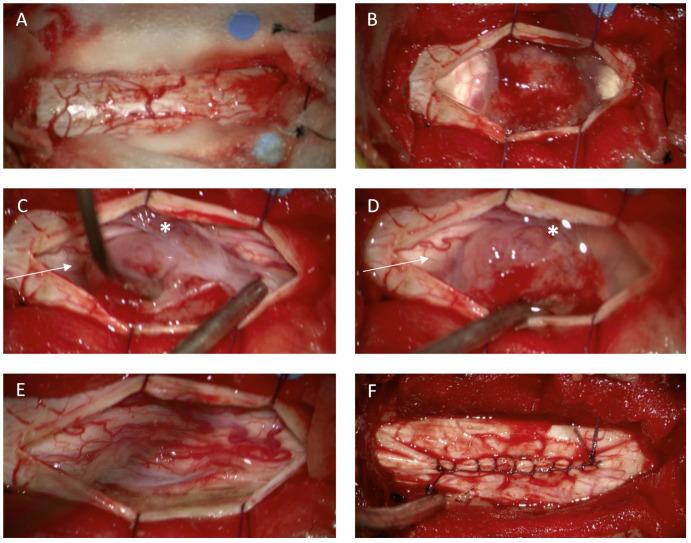
Illustrative case of a spinal meningioma. Intraoperative view in thoracic level (Th 5/6) relative to the spinal dura after removal of the vertebral arches Th 5 and Th 6 (laminoplasty approach) (**A**). spinal meningioma located ventrolaterally to the spinal cord (**B**). Dentale ligaments (**C**,**D** *) sectioned to mobilize the spinal cord and to obtain additional space for tumor removal. Spinal cord after tumor removal (**E**). The dorsal dural attachment of the tumor has been extensively coagulated (**E**). Dura closure with running stiches (**F**).

Neurosurgical removal was performed in all presented cases under microsurgical conditions. Intraoperative monitoring with somato-sensory evoked potentials and motor evoked potentials was implemented in our hospital in 1989 [19]. Mono- and bi-segmental laminectomy and hemilaminectomy were usually performed during the first years of this study [11]. This strategy was changed over the years and laminoplasty became our standard approach [20]. Details regarding ventrally/ventrolaterally located spinal meningiomas were described previously [21]. The tumor was usually removed in a piecemeal manner beginning from the center to the well-defined borders and the surrounding spinal cord tissue in order to minimize spinal cord irritation. Intraoperative neurophysiological monitoring (IONM) using MEP and SSEP was used in all cases except for emergency intervention. The surgeons were notified about any changes in amplitude and latency of the MEP and SSEP. A resection of spinal meningioma corresponding to Simpson grade I or II [14] was defined as complete according to intraoperative observation and postoperative MRI (Figure 1 and Figure 2). According to the surgical reports, the degree of calcification was defined as complete, partial, or absent. Neurological recovery and deterioration were defined as an increase or a decrease of at least one point on the mMCS, respectively.

### 2.4. Statistical Analyses

For all statistical analyses, we used SPSS 26 (IBM Corporation, Armonk, NY, USA). Univariate analyses were performed to determine predictors of poor outcome at the first and last follow-ups. For dichotomized variables, the χ^2^ test (sample size > 5) or Fisher’s exact test (sample size ≤ 5) were used. Continuous variables were analyzed using the Student’s *t*-test (normally distributed data) or the Mann–Whitney U test (non-normally distributed data). Kendall’s tau-b was assessed for continuous and ordinal variables; Spearman’s rho was used for continuous and dichotomous variables. Results were considered statistically significant at a *p*-value of <0.05. Variables with *p*-values < 0.1 were ultimately assessed in a multivariate analysis using binomial logistic regression. Kaplan–Meier curves were plotted for the entire cohort and stratified by implementation of statistically significant factors leading to a functional recovery assessed through the mMCS. The log-rank test was used to compare the cumulative recovery distribution of stratified factors seen in the Kaplan–Meier curves. Data were censored if patients were lost to follow-up.

## 3. Results

### 3.1. Patient Cohort

A total of 300 patients were included in the analysis: 156 in the HC and 144 in the CC. The incidence of 7.6 patients/year in the HC was almost double that in the CC (14.4 patients/year). The mean age was 64.7 ± 12.8 and 61.1 ± 15.3 years in the HC and CC, respectively. The number of patients under 50 years of age was 8.5% in the HC versus 21.1% in the CC. The male-to-female ratio was 1:7.2 in the HC and 1:5.5 in the CC. Cardiovascular diseases were the most frequently observed previous diseases according to the CCI (HC: 4%, n = 12, CC: 6.3%, n = 19). Detailed cohort characteristics are summarized in Table 1.

### 3.2. Tumor Location and Symptoms

The thoracic spine was the most common location in both cohorts (combined: 68.1%, n = 204) followed by the cervical spine (23.7%, n = 71). Regarding the relation to the spinal cord, we subclassified ventrally/ventrolaterally (49.3%) and dorsally/dorsolaterally (50.7%) located meningiomas. Detailed information is listed in Table 1.

Exposing symptoms were dependent on level of lesion in the spine; thoracic meningiomas mainly caused paraparesis, sensory disturbances, and myelopathy symptoms, while patients with thoracolumbar and lumbar meningiomas exhibited pain/lumbago as a typical first symptom. Extensive information is listed in Table 2.

### 3.3. Functional Outcome

In the HC, the mean follow-up was 38.0 ± 44.2 months (n = 68 patients at this point). In the CC, the mean follow-up was 25.7 ± 32.2 months including 65 patients at this point. In the total cohort, the first follow-up was 7.7 ± 0.7 months, to which 20.3% of patients were lost. The last follow-up was 49.7 ± 2.8 months; 35.6% of patients were lost to this follow-up.

In patients < 50 years of age, the mean duration from the beginning of the symptoms until surgery was 5.8 ± 3.7 months, while it was 7.7 ± 7.8 months for those aged >50 years. The mean time interval from the occurrence of first symptoms until surgery amounted to 8.8 ± 8.5 months in the HC and 6.1 ± 5.7 months in the CC.

**Figure 3:**
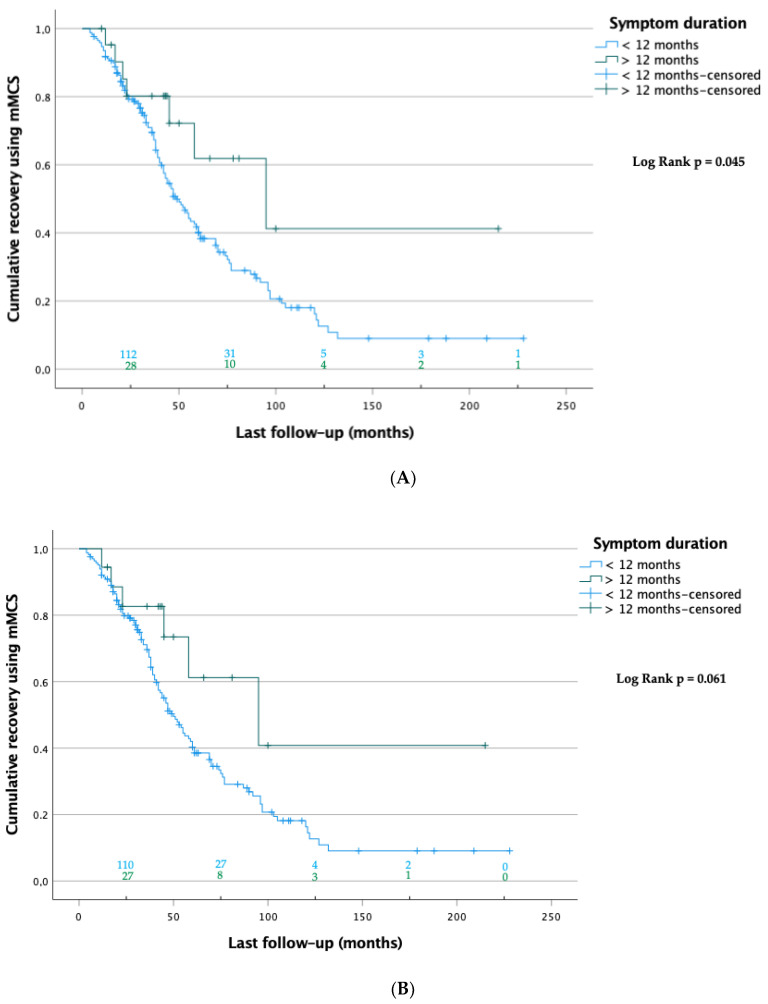
(**A**) **Kaplan-Meier curves of patients at last FU (n = 193).** Data were censored when patients were lost to follow up at this point. The numbers of patients at risk are shown on top of the abscissa of each group. The log-rank test showed significant differences between the groups. (**B**) **Sensitivity** **analysis:** Kaplan–Meier curves of patients with first and last follow-ups (n = 183). Data were censored when patients were lost to follow-up at this point. The numbers of patients at risk are shown on top of the abscissa of each group. The log-rank test showed no significant differences between the groups.

The Kaplan–Meier analysis showed that at last follow-up, patients with a symptom duration < 12 months recovered significantly faster than those with a symptom duration > 12 months (70.1 ± 5.8 vs. 121.5 ± 27.1 months, respectively; *p* = 0.045; Figure 3A). The sensitivity analysis, which considered only patients who were followed up twice, showed a trend toward significance (70.4 ± 5.9 vs. 121.4 ± 28.2 months, respectively; *p* = 0.061; Figure 3B). The same analysis also confirmed that regarding poor outcomes, patients who dropped out between the first and last follow-up did not significantly differ from those who were followed up (mMCS > 3) (46.4% vs. 58.5%, respectively; *p* = 0.126; Table 3).

Multivariate analysis using binomial logistic regression including age, preoperative CCI, location of the tumor, operation period, and expansion of the lesion identified ventral location of the tumor relative to the spinal cord as an independent predictor of poor outcome (mMCS > 3) at first follow-up (Table 4).

**Figure 4:**
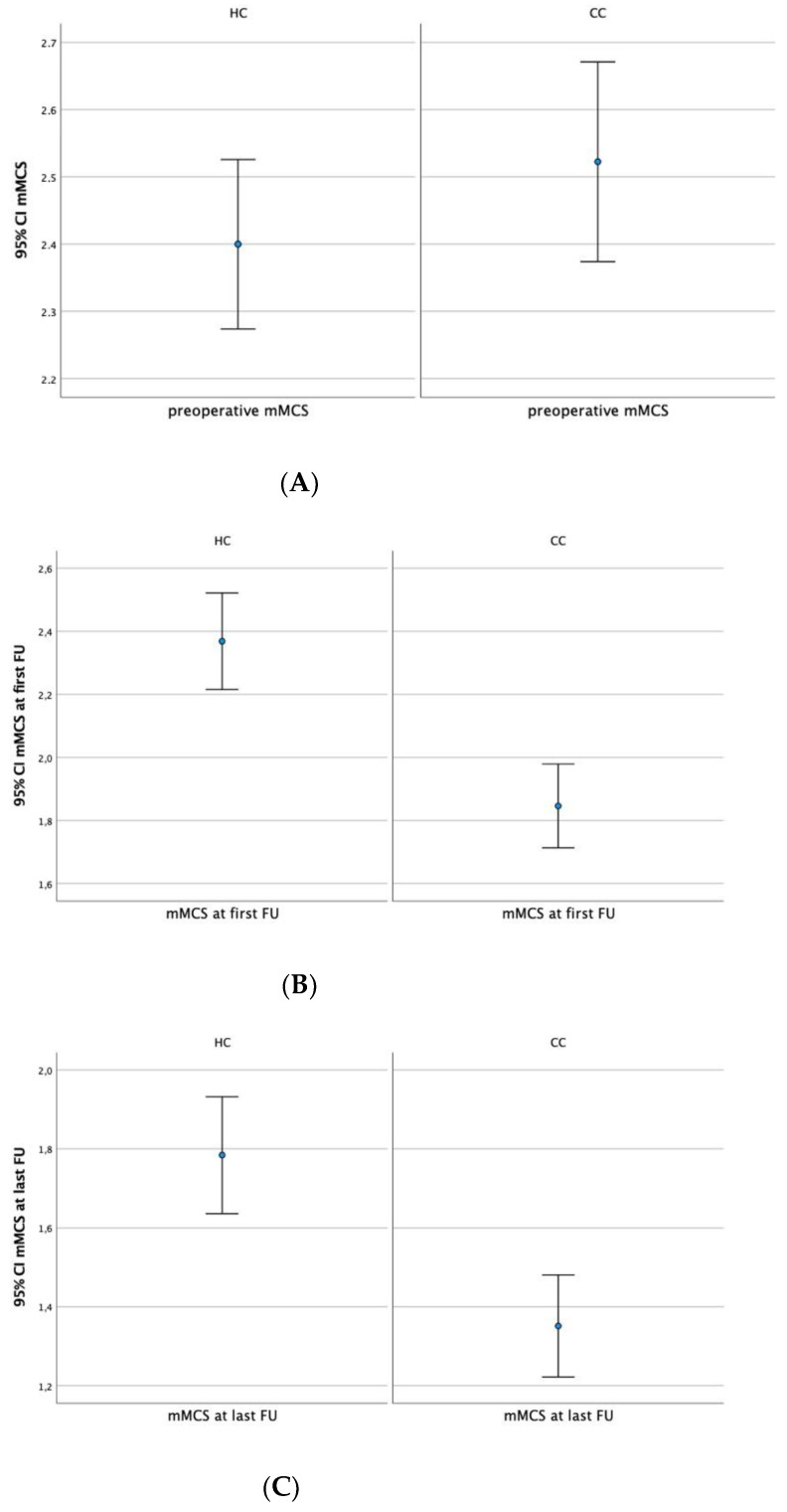
(**A**–**C**). Comparison of the preoperative (**A**) and postoperative (**B**,**C**) mMCS between HC and CC showing statistically significantly better neurological outcomes at first (**B**) and last follow-ups (**C**) in the CC than in the HC.

The preoperative neurological status was not different between the HC and CC regarding the area of surgical treatment (mMCS, 2.4 ± 0.7 vs. 2.5 ± 0.9, respectively; *p* = 0.294; Figure 4A). However, neurological outcomes at the first and last follow-ups were significantly improved in the CC compared with the HC (first follow-up: mMCS, 1.9 ± 0.7 vs. 2.4 ± 0.9, respectively; last follow-up: mMCS, 1.4 ± 0.6 vs. 1.8 ± 0.8, respectively; *p* < 0.01 for both; Figure 4B,C). Furthermore, a significant decrease in symptom duration was observed between the CC and HC (6.1 ± 5.7 vs. 8.8 ± 8.5 months, respectively; *p* = 0.034).

### 3.4. Complications

Postoperative complications were identified in 13 patients in the combined cohort (4.3%), including postoperative epidural hemorrhage in 3 cases, postoperative cerebrospinal fluid fistula in 5 cases, necessity of an external ventricular drain in 1 case of bifrontal intracranial air trapping after operation in a semi-sitting position, and superficial wound infection in 4 cases. Despite the temporary use of external ventricular drain and superficial surgical debridement, no further surgical interventions were required. The frequency distribution was similar in both cohorts (HC: 4.5%, n = 7; CC: 4.2%, n = 6).

### 3.5. Histological Findings

Histological examinations confirmed meningiotheliomatous meningioma in 108 cases (HC: 25%, CC: 47.9%), psammomatous meningioma in 66 (HC: 22.4%, CC: 21.5%), transitional meningioma in 60 (HC: 19.9%, CC: 20.1%), and fibroblastic meningioma in 42 (HC: 19.2%, CC: 8.3%). Endotheliomatous meningioma was diagnosed in 16 patients (HC: 10.3%, CC: 8.5%), metaplastic meningioma in 3 (HC: 1.3%, CC: 0.7%), angiomatous meningioma in 2 (HC: 0.6%, CC: 0.7%), and secretory meningioma in 1 (HC: 0%, CC: 0.7%). In the entire cohort, two cases were classified as grade Ⅱ according to the WHO classification (HC: 1.3%, CC: 0%), none as grade III, and the remainder as grade I (HC: 98.7%, CC: 100%) [22].

### 3.6. Recurrence

Reoperation due to recurrence was necessary in four cases (three cases in the HC and one case in the CC) due to infiltrative growth after Simpson grade II resection in ventrally located spinal meningioma (WHO grade I). Detailed information is available in Table 5.

## 4. Discussion

In this study, we analyzed our results for over three decades of spinal meningioma surgery to identify potential differences over time that could be attributed to technical advances. Our results were comparable with those of several studies on the surgical management of spinal meningiomas [11,23,24,25]. In general, the clinical outcome of surgically treated patients with spinal meningioma is good. We found that a shorter interval between the occurrence of the first symptoms and surgery led to a swifter neurological improvement in the long term. At the same time, in the past decade, affected patients have been diagnosed earlier and at a younger age as compared to before 2008. Nevertheless, the literature also describes cases without improvement in neurological outcome after surgical treatment or even postoperative deterioration in rare cases [1,26,27]. Reasons for absent neurological recovery are discussed controversially. Histological characteristics, tumor localization, partially resected lesions, and duration from the first symptoms until surgery are candidates for important predictors associated with clinical outcomes [10,25,28,29]. In this study, our experience over 30 years with 300 patients was presented with special emphasis on two different periods of time.

### 4.1. Patient Cohort

The majority of patients in our study were female (entire cohort: 86.3%; HC: 87.8%; CC: 84.7%). This was in line with the findings of Narayan et al. [1], which showed that female sex accounts for 75–90% of spinal meningioma cases. This was based on sex hormones, as the level of estrogen in patients’ blood serum as well as the hormone-receptor status in the tumor cells were significant factors associated with the occurrence of meningioma in females [30,31]. Whilst spinal meningiomas most frequently affect middle-aged patients [5], which was confirmed in our complete cohort, the mean age was slightly lower in the CC (61.1 ± 15.3 years) as compared to the HC (64.7 ± 12.8 years). Cramer et al. [8] detected that old age was a risk factor for poor outcomes [7,8,9]. We could not confirm this in our study, neither in the HC nor in the CC. In contrast, we found that patients in the CC were significantly younger, and the number of patients under 50 years of age doubled relative to the HC.

One of the main reasons for the increased cases of spinal meningiomas in younger patients is probably the better accessibility to MRI over the decades [32,33]. This has led to an increase in diagnostic imaging, resulting in a greater number of diagnostic findings in less time. While in 2005 only 1640 MRI units were available in Germany, producing a total number of 6,003,944 (6,003,944) MRI examinations stationary and ambulatory, 2840 MRI units were accessible in 2016, leading to around 11,812,067 stationary and ambulatory MRI examinations [34]. This increase in MRI examinations and its consequences were analyzed by Lurie et al. [35] and Verrilli et al. [36], who detected a simultaneous increased number of MRI examinations and an increased number of operations. Similarly, Klekamp and Samii [10] documented that the emergence of MRI enabled an earlier diagnosis and a shorter period of symptoms until the correct diagnosis and therapy. These developments could also be correlated significantly with a better neurological outcome. Additionally, the increased caseload in the CC may have had an impact on surgical treatment and outcome due to improved surgical routines.

On the other hand, age-related comorbidities such as degenerative spinal diseases, diabetes mellitus, and polyneuropathy, as well as the reduced physical constitution covering the symptoms of spinal meningiomas, may result in a later diagnosis, including later MRI examinations, in older patients.

### 4.2. Tumor Location

Whilst we most commonly detected thoracic spinal meningiomas in this study in the HC as well as in the CC, a cervical location was found less frequently, and lumbar meningiomas were only observed in a small subset of patients. These findings were consistent with previous studies [9,10,28]. We subclassified meningiomas according to their ventrolateral and dorsal location relative to the spinal cord; our findings, particularly on tumor location, confirmed past studies [10]. Ventrally located meningiomas correlate with a poorer prognosis [6,37]. We could confirm this observation only at the first follow-up, indicating that our patients could recover well in the long term even if the tumor was located ventrally. Additionally, we could verify that an earlier diagnosis and therapy of spinal meningiomas led to a better neurological outcome. As ventral spinal meningiomas lack specific symptoms or develop nonspecific symptoms such as sensory disorders in the early phase, they are difficult to confirm [38,39]. This leads to a long duration of symptoms until the correct diagnosis and therapy, and irreversible damage of the spinal cord ensues. This may explain why we did not observe a better functional outcome after surgical therapy of ventrally located spinal meningiomas in the first seven months after surgery.

### 4.3. Surgical Outcome

In our study, Simpson grade II resections were achieved in 79% (122 cases in the HC and 115 cases in the CC) and Simpson grade I resections in 12.3% (19 cases in the HC and 18 cases in the CC) of all cases. This corresponded to the known rates of complete tumor removal, which range between 82% and 98% [2,7]. Half of the incompletely resected spinal meningiomas showed massive calcification; this could explain a subtotal removal and the implied higher risk for irreversible damage and complications if a complete resection is enforced [12,40].

The aim of the surgical therapy of spinal meningiomas is total tumor resection with minimized spinal cord manipulation [41]. Whereas laminectomy was preferred in most of the cases in the HC, laminoplasty was performed most frequently in the CC. Interestingly, we did not observe secondary stabilization during in-patient stay or follow-up due to surgical therapy of the spinal meningioma. Laminoplasty is recommended as the standard approach for spinal pathologies because it is associated with a shorter hospital stay and postoperative improvement according to the Nurick scale and modified Japanese Orthopaedic Association scale [1,28,41,42]. This is the current standard approach in our clinic as well. Individual decision regarding localization of the pathology is important because laminoplasty usually can be conducted in tumors that can be extirpated without facetectomy [14,25]. However, in cases in which the reinsertion of the vertebral arch and fixation with miniplates and screws were not safe enough, as well as in cases with advanced osteoporosis, laminectomy was performed. Despite the modification of the surgical technique over time (more laminoplasty than laminectomy), patients’ overall outcome remained proficient.

### 4.4. Functional Outcome

The preoperative neurological outcome did not differ between the two cohorts. However, the postoperative mMCS at first and last follow-up was significantly better in the CC compared to the HC (*p* < 0.01). One reason was that an earlier diagnosis and a resulting shorter symptom duration led to a significant improvement in the postoperative neurological outcome compared to symptom duration >12 months (*p* = 0.045). Solero et al. [9] were also able to show the importance of an early diagnosis for a better functional outcome in their study. Nevertheless, complete neurological recovery after surgery and rehabilitation was detected even in patients with severe preoperative neurological deficits in both cohorts equally. Therefore, surgery should be performed in any case to prevent ongoing deterioration and to enable postoperative recovery. This finding was in line with the results of King et al. [41], who described patients with paraplegia or bladder dysfunction who were independently mobile or showed normal bladder function after surgery and rehabilitation.

### 4.5. Histological Findings

Our study confirmed the most common histological features of spinal meningiomas, including meningiotheliomatous, psammomatous, transitional, and fibroblastic meningiomas, in both cohorts. Furthermore, given our relatively large sample size, we observed endotheliomatous, metaplastic, and secretory meningiomas. Malignancy aggravated prognosis as already described previously with regard to the functional outcome [9,13,38].

### 4.6. Recurrence

Tumor recurrence was found in four cases. Klekamp and Samii [10] stated that en plaque and infiltrative growing spinal meningiomas with arachnoid scarring showed significantly higher recurrence rates [10]. Our analyses affirmed these reports, thus warranting a re-evaluation of surgical techniques in spinal surgery [42].

### 4.7. Limitations of the Study

The retrospective design of our study was associated with inherent bias and bore the risk of incomplete data with additional limitation and selection bias. Although our study assessed a large cohort over a long period of time, it only represented results of a single center and was not population-based. During the long study period, some surgical techniques were changed. The patients were treated by different neurosurgeons, which could be another confounding factor. In addition, the lower image quality of MRI in the initial phase of this study must be considered because it might have influenced the quality of the data. Prospective multicenter studies are urgently needed to confirm our findings and to assess further predictors of patient outcome. Further analyses that include examination of the current radiological findings after spinal tumor removal are planned in our clinic to identify postoperative complications or instabilities that require preventive stabilization.

## 5. Conclusions

Surgical therapy of spinal meningiomas remains the gold standard that allows a recovery in all patients independent of their preoperative mMCS. A shorter symptom duration (<12 months) leads to a significant better postoperative neurological improvement at last follow-up.

## Figures and Tables

**Table 1:** Patient and surgical characteristics summarized.

Characteristicstics	Historical Cohort	Current Cohort	Total
Period of time	1990–2007	2008–2020	1990–2020
Number of patients	156	144	300
Age (years), mean ± SD	64.7 ± 12.8	61.1 ± 15.3	63.1 ± 14.0
Number of patients <50 years	17 (9.0%)	29 (20.1%)	43 (14.3%)
Sex (n, %)			
Male	19 (12.2%)	22 (15.3%)	41 (13.7%)
Female	137 (87.8%)	122 (84.7%)	259 (86.3%)
Level of the Spine (n, %)			
Cervical	30 (19.2%)	41 (28.5%)	71 (23.7%)
Cervicothoracic	8 (5.1%)	2 (1.4%)	10 (3.3%)
Thoracic	112 (71.8%)	92 (63.9%)	204 (0.68%)
Thoracolumbar	2 (1.3%)	1 (0.7%)	3 (0.7%)
Lumbar	4 (2.6%)	8 (5.6%)	12 (4.2%)
Tumor location (n, %) (in relation to spinal cord)			
Ventrally	22 (14.1%)	42 (29.2%)	64 (21.3%)
Ventrolaterally	44 (28.2%)	40 (27.8%)	84 (28.0%)
Dorsally	58 (37.2%)	39 (27.1%)	97 (32.3%)
Dorsolaterally	32 (20.5%)	23 (16.0%)	55 (18.3%)
Surgical Approach (n, %)			
Laminectomy	106 (67.9%)	46 (31.9%)	152 (50.7%)
Hemilaminectomy	11 (7.1%)	13 (9.0%)	24 (8.0%)
Laminoplasty	39 (25%)	85 (59%)	124 (41.1%)
Duration from the first symptoms to surgery (months, mean ± SD)	8.8 ± 8.5	6.1 ± 5.7	7.4 ± 7.3
Resection À Simpson grade (n, %)			
Grade 1	19 (12.2%)	18 (12.5%)	37 (12.3%)
Grade 2	22 (78.2%)	115 (79.9%)	237 (79.0%)
Grade 3	12 (7.7%)	7 (4.9%)	19 (6.3%)
Grade 4	3 (1.9%)	4 (2.8%)	7 (2.3%)
Tumor adhesion (n, %)	47 (30.13%)	33 (22.9%)	80 (26.67%)

**Table 2:** Patients’ first symptoms.

Presenting	Cervical	Cervicothoracic	Thoracic	Thoracolumbar	Lumbar
Symptoms	(n, *p*-Value)	(n, *p*-Value)	(n, *p*-Value)	(n, *p*-Value)	(n, *p*-Value)
Pain/Lumbago	20 (0.07)	4 (0.063)	84 (0.042 *)	3 (<0.01 *)	12 (<0.01 *)
Sensory disorder ^a^	40 (0.026 *)	5 (0.05)	196 (<0.01 *)	1 (0.217)	4 (0.078)
Motoric deficits ^b^	37 (0.02 *)	6 (0.03 *)	185 (<0.01 *)	--	--
Myelopathy	--	2 (0.062)	71 (0.025 *)	--	--

**^a^** Sensory disorder was defined as hypesthesia, paresthesia, or dysesthesia. **^b^** Motoric deficits were defined as monoparesis arms, monoparesis legs, paraparesis, or tetraparesis. * Illustrates significant *p*-value (<0.05).

**Table 3:** **Sensitive analysis of dropouts between the first and the last follow-ups** confirming that there was no statistically significantly differences from the followed patients regarding poor outcome.

	Value	df	Asymptomatic Significance	Exact Sig	Exact Sig	Point
			(2-Sided)	(2-Sided)	(1-Sided)	Probability
Pearson Chi-Square	2.519	1	0.112	0.126	0.076	
Continuilty Correction	2.055	1	0.152			
Likelihood Ratio	2.506	1	0.113	0.126	0.076	
Fisher’s Exact Test					0.126	0.076
Linear-by-Linear Association	2.508	1	0.113	0.126	0.076	0.035
N Valid Cases	239					

**Table 4:** Univariate analyses and multivariate binomial logistic regression regarding outcomes at first and last follow-ups. * Illustrates significant *p*-value (<0.05).

			Outcome at First Follow-Up		Outcome at Last Follow-Up	
			*p*		*p*	
***Mann-Whitney U Test***						
Age			0.012 *		0.010 *	
CCI			0.116		0.894	
Symptom duration			0.501		0.303	
***Cochran-Armitage Test for Trend***						
Expansion of the lesion (1/2/>2 segments)			0.915		0.084	
Level of the spine (C/Th/L)			0.756		0.355	
Grade of resection			0.841		0.901	
***Chi-Square Test***						
Sex			0.368		1.000	
Location (ventral vs. dorsal)			0.028 *		0.241	
Surgical approach			0.173		0.629	
(Laminectomy vs. Laminoplasty vs.Hemilaminectomy)						
Tumor adhesion			0.525		1.000	
Postoperative complications			0.646		1.000	
Operation period (HC vs. CC)			0.060		0.499	
**Multivariate Analysis-*Binomial Logistic Regression***						
			**Outcome at First Follow-Up**		**Outcome at Last Follow-Up**	
**Predictors**	**Exp (B)**	***p***	**95% CI**	**Exp (B)**	***p***	**95% CI**
Age	1.060	0.053	0.999–1.124	1.336	0.083	0.963–1.853
Location (ventral vs. dorsal)	6.076	0.025 *	1.254–29.452	--	--	--
Operation period (HC vs. CC)	3.963	0.089	0.811–19.369	--	--	--
Expansion of the lesion (1/2/>2 segments)	--	--	--	4.085	0.265	0.345–47.661

**Table 5:** Characteristics of Recurrent Operations (n).

Characteristicstics	Historical Cohort	Current Cohort	Total
Level of the Spine			
Cervical	1	1	2
Cervicothoracic	1	0	1
Thoracic	1	0	1
Tumor location (in relation to spinal cord)			
Ventrolaterally	3	1	4
Surgical Approach			
Laminectomy	2	0	2
Hemilaminectomy	1	0	1
Laminoplasty	0	1	0
Resection À Simpson grade			
Grade 2	3	1	4
WHO-Grade	3	1	4
I			
Infiltrative growing (n, %)	3	1	4
Time of recurrent operation after first surgery			
2 years	0	1	1
3 years	1	0	1
10 years	2	1	2

## Data Availability

Not applicable.
